# Postpartum cessation of urban space use by a female baboon living at the edge of the City of Cape Town

**DOI:** 10.1002/ece3.9963

**Published:** 2023-05-16

**Authors:** Anna M. Bracken, Charlotte Christensen, M. Justin O'Riain, Ines Fürtbauer, Andrew J. King

**Affiliations:** ^1^ School of Biodiversity One Health and Veterinary Medicine Graham Kerr Building Glasgow G12 8QQ UK; ^2^ Biosciences, School of Biosciences, Geography and Physics, Faculty of Science and Engineering Swansea University SA2 8PP Swansea UK; ^3^ Department of Evolutionary Biology and Environmental Studies University of Zürich Winterthurerstrasse 190 8057 Zürich Switzerland; ^4^ Department of Biological Sciences, Institute for Communities and Wildlife in Africa University of Cape Town Cape Town South Africa

**Keywords:** anthropogenic environments, birth, GPS, life history

## Abstract

Species with slow life history strategies that invest in few offspring with extended parental care need to adapt their behavior to cope with anthropogenic changes that occur within their lifetime. Here we show that a female chacma baboon (*Papio ursinus*) that commonly ranges within urban space in the City of Cape Town, South Africa, stops using urban space after giving birth. This change of space use occurs without any significant change in daily distance traveled or social interactions that would be expected with general risk‐sensitive behavior after birth. Instead, we suggest this change occurs because of the specific and greater risks the baboons experience within the urban space compared to natural space, and because leaving the troop (to enter urban space) may increase infanticide risk. This case study can inform methods used to manage the baboons' urban space use in Cape Town and provides insight into how life history events alter individuals' use of anthropogenic environments.

## Graphical abstract

Using high‐resolution GPS data we show that a female chacma baboon (*Papio ursinus*) who commonly ranges within urban spaces in the City of Cape Town, South Africa, stops using urban space after giving birth. We suggest this change occurs because of the risks the baboons face when leaving the troop and using urban space. This case study will be important for baboon management in the region, and provides insight into how life history events alter individuals' use of anthropogenic environments.

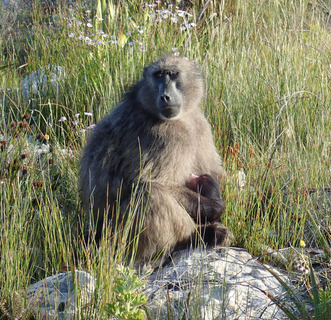

Species with slow life history strategies typically have few offspring and high investment in both social relationships and parental care. Consequently species with slow life history strategies are less able to compensate for increased mortality rates (Purvis et al., 2000), including mortality associated with human disturbance (Quetglas et al., 2016; Wheeler et al., 2020). For example, fishing affects slow‐strategy species (elasmobranchs) but not fast‐strategy species (cephalopods), due to differences in generation times and population turnover (Quetglas et al., 2016). For threatened carnivore and primate species, a long gestation is correlated with higher extinction risk (Purvis, 2001) which may be exacerbated for animals using human‐modified landscapes that are subject to rapid changes in the selective environment (Palumbi, 2001). Therefore, species with slow strategies may have to adjust their behavior within their lifetime (Sih et al., 2011) to survive in human‐modified landscapes, which often manifests as crop‐ or urban‐foraging behavior (Bateman & Fleming, 2012; Chiyo et al., 2012; Fehlmann et al., 2021; McLennan et al., 2017; Moss et al., 2016; Warren et al., 2007).

Chacma baboons (*Papio ursinus*) are a typical slow life history species, living in cohesive mixed‐sex social groups with strong social relationships (Cheney et al., 2016; Silk et al., 2010). Female baboons show high maternal investment (Altmann & Samuels, 1992) giving birth to a single infant after a gestation period of 6 months (Altmann et al., 1977; Archie et al., 2014), carrying the infant for several months after birth (Altmann & Samuels, 1992), and lactating for up to a year (Archie et al., 2014; Zipple et al., 2017). Mothers reduce activity levels in the first few months after birth (Barrett et al., 2006) because of both high energetic costs due to lactation (Altmann, 1983; Barrett et al., 2006) and the risk of infanticide by adult males (Zipple et al., 2017). As is the case with other primates (Dunayer & Berman, 2018), new mothers receive increased social attention as other individuals inspect and handle their offspring (Altmann, 2001; Henzi & Barrett, 2002).

Chacma baboons living at the edge of the City of Cape Town, South Africa, frequently use urban spaces (Bracken et al., 2021; Fehlmann, O'Riain, Kerr‐Smith, Hailes, et al., 2017), resulting in high levels of spatial overlap and negative interactions with people (Hoffman & O'Riain, 2012). Human residents and land‐owners often experience baboon‐caused damage to their property and economic losses (e.g., when baboons forage in commercial vineyards) (Fehlmann, O'Riain, Kerr‐Smith, Hailes, et al., 2017; Kaplan et al., 2011; van Doorn & O'Riain, 2020). Consequently, residents harbor negative attitudes toward baboons (Mormile & Hill, 2017), and baboons experience high levels of human‐induced mortality and injury (Beamish, 2009). While urban spaces in Cape Town can provide baboons with higher energy foods (Fehlmann et al., 2021; Fehlmann, O'Riain, Kerr‐Smith, Hailes, et al., 2017; Hoffman & O'Riain, 2012; Kaplan et al., 2011) they are also more costly than other habitats because of increased activity demands and risks of being “herded” away from urban space by the employed “baboon rangers” (Fehlmann, O'Riain, Kerr‐Smith, Hailes, et al., 2017; Fehlmann, O'Riain, Kerr‐Smith, & King, 2017). To exploit foraging opportunities presented by urban space while avoiding detection by the rangers, baboons living at the edge of the City of Cape Town therefore tend to make short visits to urban space alone or in small groups (Bracken et al., 2021; Fehlmann, O'Riain, Kerr‐Smith, Hailes, et al., 2017).

During the study of a baboon troop (*n* = 21 adults; 2 males and 19 females) that ranges within and on the periphery of the Da Gama Park suburb of Cape Town, several females gave birth (Figure 1), one of which (a low‐ranking [ranked 13 out of 19 females] female baboon: F13) was wearing a GPS‐equipped tracking collar (collars were deployed on *n* = 16 adults [Bracken et al., 2021; Bracken et al., 2022; McCann et al., 2021]; see Appendix). The birth occurred 32 days into the time the female's GPS was recording (she was confirmed as “with infant” on 03/09/2018), with a further 16 days of GPS data collected after birth (after which her GPS stopped recording continuous data). These GPS data provided an opportunity to examine potential changes in this female's use of natural and urban space. By creating a polygon around the urban/natural space in QGIS (QGIS.org, 2020), we calculated the time the female spent in urban space using the package “Recurse” in R (Bracis et al., 2018), and created a 95% home range (estimated using fixed kernel densities: Calenge, 2006), based on movements before and after birth. Additionally, since giving birth can reduce activity levels in new baboon mothers (Barrett et al., 2006), we calculated daily distance traveled before and after parturition using the function “as.ltraj” in the “adehabitat” package in R (Calenge, 2006). Lastly, because giving birth is associated with changes in social interactions (Alberts, 2019; Seyfarth, 1978), we estimated individual strength (Morrow et al., 2019; Peignier et al., 2019; Silk et al., 2003) and eigenvector centrality (Bracken et al., 2021; Morrow et al., 2019) based on a network of associations (proximity‐based using the package “Spatsoc” in R, see: Bracken et al., 2021, 2022) before and after birth, for collared individuals with overlapping data for both time periods (*n* = 11 individuals). At the time of our study, the troop contained two adult males (the alpha: M1, and the beta: M2) with an unstable dominance relationship (Bracken et al., 2021). The beta male attacked a (non‐collared) female's infant during the study period with the infant dying 10 days later. For further details on our analyses, see Appendix.

**FIGURE 1:**
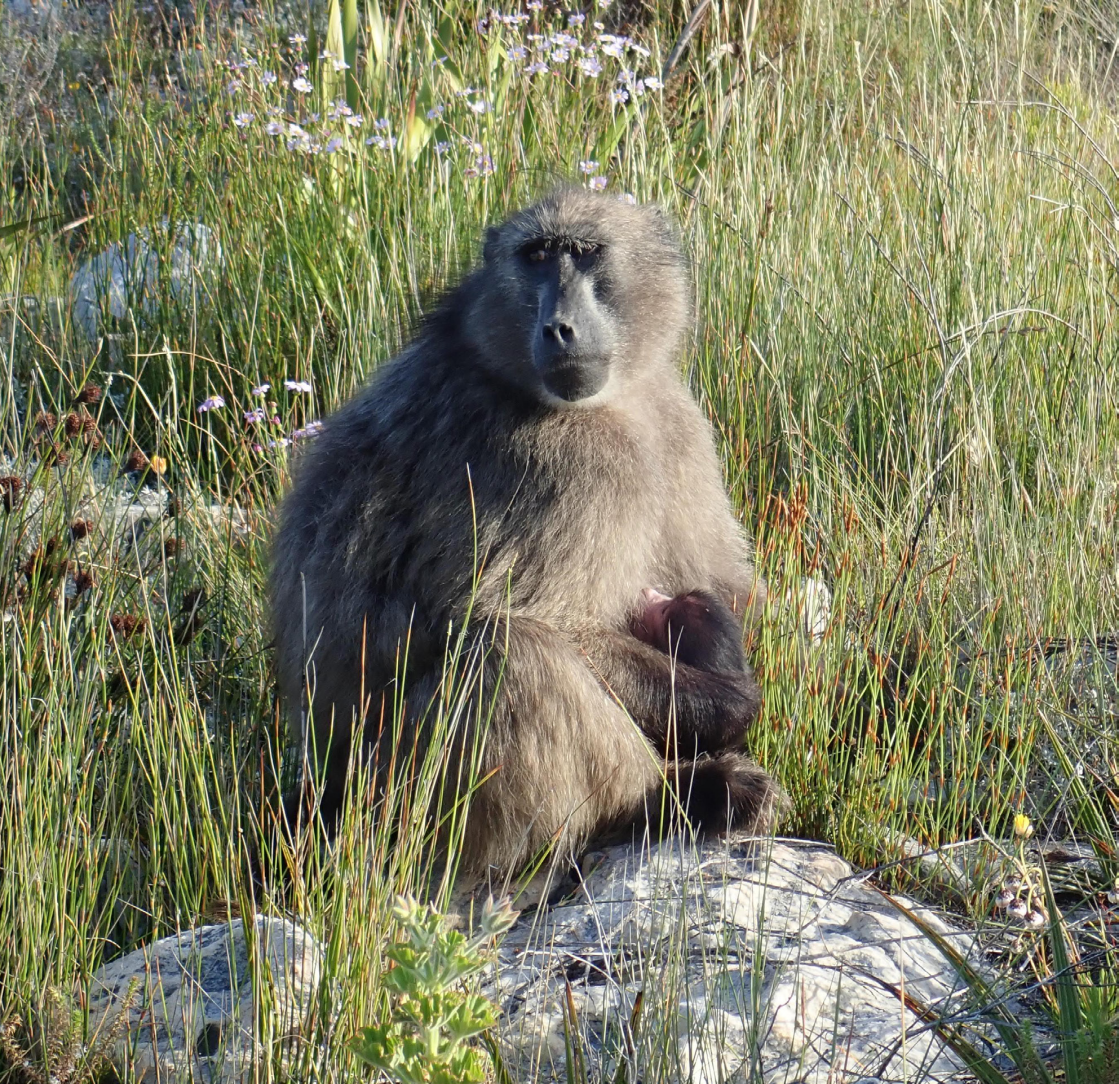
A female baboon with her newborn infant (black natal fur, pink face) from the Da Gama troop, in Cape Town, South Africa. Picture credit: Anna M. Bracken.

We found that the female baboon stopped using urban space after birth (Figure 2a) while other individuals continued to do so (Figures A1 and A2). A similar result was obtained when randomly sub‐sampling the “before birth” period and analyzing the same number of GPS days (*n* = 16) before and after birth (Figure A3). The female's daily travel distances were not affected (Figure 2b), though her home range after birth reflected the noticeable absence of urban space use (Figure 2c). The female's association patterns within the troop were similar before and after birth, and while her relative strength and centrality scores increased in the period after birth, this increase in strength and centrality was seen across all individuals (Figure 2d,e).

**FIGURE 2:**
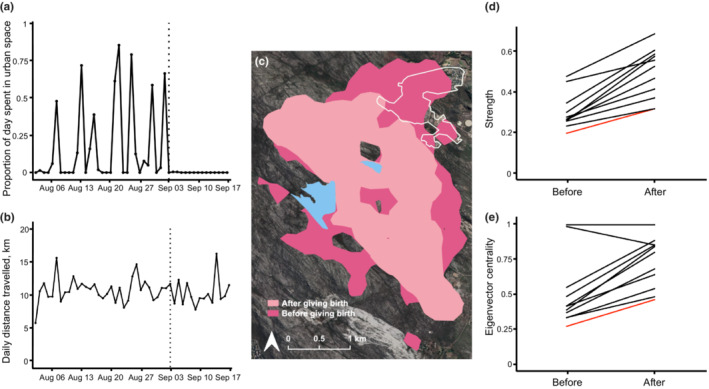
Female baboon movement and behavior before and after giving birth. (a) Proportion of the day (08:00–18:00) spent in the urban space by the female baboon across the study period. (b) Daily distance traveled (km) by the female baboon across the study period. For (a) and (b) date of birth (03/09/2018) is indicated as dashed line. (c) 95% home range of the female baboon before (dark pink) and after (light pink) giving birth (estimated using fixed kernel densities: Calenge, 2006). Urban polygon is indicated by a solid white line and dams are represented by light blue areas. (d) Individual baboon strength in proximity network based on *n* = 11 individuals. (e) Individual baboon eigenvector centrality in proximity network based on *n* = 11 individuals. For (d) and (e) focal female data shown in red.

To our knowledge, this is the first documented evidence of cessation of urban space use by an animal after giving birth. Because this female's change of urban space use occurs without any significant change in distance traveled or social association patterns, it is unlikely that the change is a consequence of a general risk‐sensitive behavior strategy after birth. Instead, it is likely that the risks specific to, and associated with, the urban space relative to natural space led to adapted movement decisions by the mother with a vulnerable infant. Though we only have GPS data for one baboon, we observed the same pattern of urban avoidance postpartum in two other non‐collared females during the study (A. M. Bracken & C. Christensen, personal observation).

In our study system, the urban space poses specific risks in the form of residents, dogs, vehicles, electric fences, and herding behavior by designated field rangers employed to reduce baboons' urban space use (Beamish & O'Riain, 2014). These risks are absent or reduced in natural space (Bracken et al., 2022; Fehlmann, O'Riain, Kerr‐Smith, Hailes, et al., 2017; van Doorn & O'Riain, 2020). Additionally, urban space use tends to cause group fragmentation, both when the whole troop is in town (Bracken et al., 2022), and when individuals split from the troop in natural space to use urban space (Bracken et al., 2021). We have previously shown in the troop studied here, that these individuals tend to be socially peripheral, low‐ranking females, which capitalize on the opportunity to use urban space (Bracken et al., 2021), as management tends to focus on adult males. Additionally, postpartum, females may be subject to infanticide risk when using urban space if they become isolated from the troop and, by extension, the protection of the alpha male (who is herded out by management). Avoidance of urban space due to infanticide from males has also been observed in female black bears (Merkle et al., 2013). The beta male in our study troop posed an infanticidal threat, and while we do not know whether the alpha or beta male sired F13's infant (which we focus on here), a high risk of infant mortality from infanticide attempts (Cheney et al., 2006; Palombit, 2003; van Doorn et al., 2010) and defense of infants by alpha males from attacks by other males (Cowlishaw, 1999) may have contributed to F13 remaining with the main troop and using natural space more after birth.

We therefore suggest that the specific risks baboons encounter in urban space as a consequence of their interaction with people (Kaburu et al., 2019) and the urban environment (Soulsbury & White, 2016) combined with group fragmentation and reduced protection of the alpha male when in urban space (Bracken et al., 2021, 2022) resulted in the female's decision to avoid urban space. This observation is important for baboon management in the region. The Cape baboon population is growing consistently (Beamish & O'Riain, in press) with numbers of individuals in managed peri‐urban troops increasing from 248 individuals in 2006 to 445 in 2020 (in TimesLive, 2020). Consequently, contraception has been proposed as a potential non‐lethal mechanism to restrict numbers within troops on the Cape (O'Riain, 2021; Richardson, 2020). While contraception has been successfully used for captive baboon species (Plowman et al., 2005), it is unclear how it would influence the behavior and movement of females who use the urban space as much as, or more than, males (Bracken et al., 2021). Indeed, our findings suggest that if females are prevented from reproducing, this could translate to even more urban space use by the City's chacma baboons. Additionally, since reproduction is fundamental for female baboons' life histories (signaling female quality: Domb & Pagel, 2001 and driving social relationships between males: Seyfarth, 1978 and other females: Seyfarth, 1976), suppression of the reproductive cycle may have a number of unintended social consequences.

We hope this study will initiate further research into how animals, which invest heavily in parental care, are responding to the benefits and risks associated with human‐modified landscapes. For slow strategy species, parental care is essential to infant survival and in this study, we suggest that the reduction in female urban space use postpartum is an adaptive behavioral response that will improve both her and her offspring's survival. A larger sample size of data for female baboons that are using urban areas before and after birth will provide important insights not only into how wildlife is responding to the Anthropocene, but also into the methods used for managing wildlife on the urban edge.

## AUTHOR CONTRIBUTIONS

**Anna M. Bracken:** Conceptualization (equal); data curation (equal); formal analysis (lead); investigation (equal); methodology (equal); visualization (lead); writing – original draft (lead). **Charlotte Christensen:** Data curation (equal); investigation (equal); writing – review and editing (equal). **M. Justin O'Riain:** Project administration (equal); writing – review and editing (equal). **Ines Fürtbauer:** Funding acquisition (equal); project administration (equal); resources (equal); supervision (supporting); writing – review and editing (equal). **Andrew J. King:** Conceptualization (equal); funding acquisition (equal); methodology (equal); project administration (equal); resources (equal); supervision (lead); visualization (supporting); writing – review and editing (equal).

## CONFLICT OF INTEREST STATEMENT

The authors declare no competing interests.

## Figures and Tables

**FIGURE A1:**
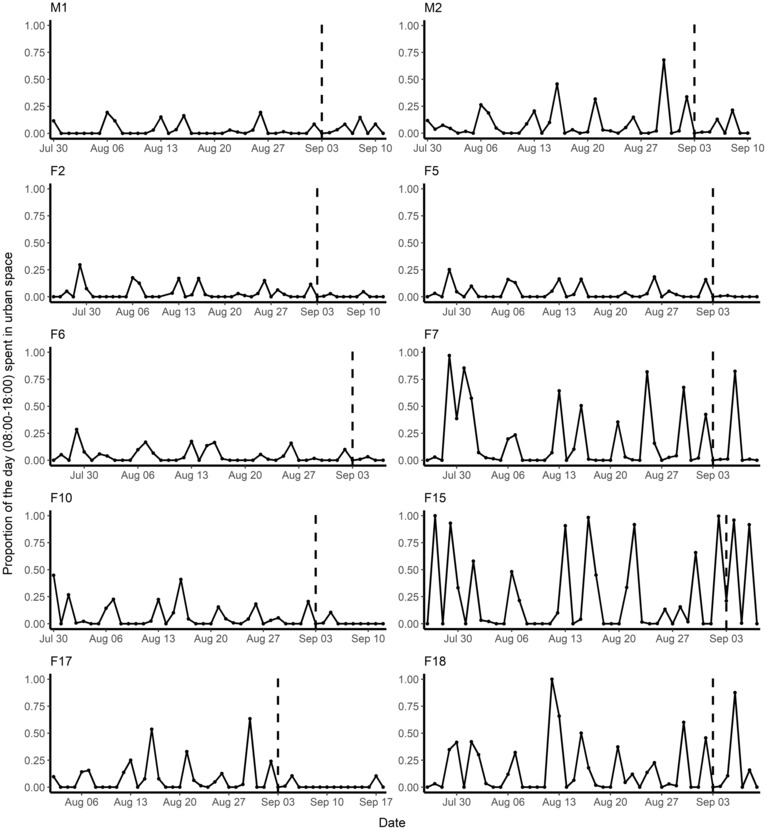
Proportion of the day (08:00–18:00) spent in urban space for each individual before and after the focal female (F13) gave birth (birth date indicated by a vertical dashed line on 03/09/2018). Start and end dates vary between individuals as collar deployment on baboons occurred over several days and individual GPS recorded for different lengths of time.

**FIGURE A2:**
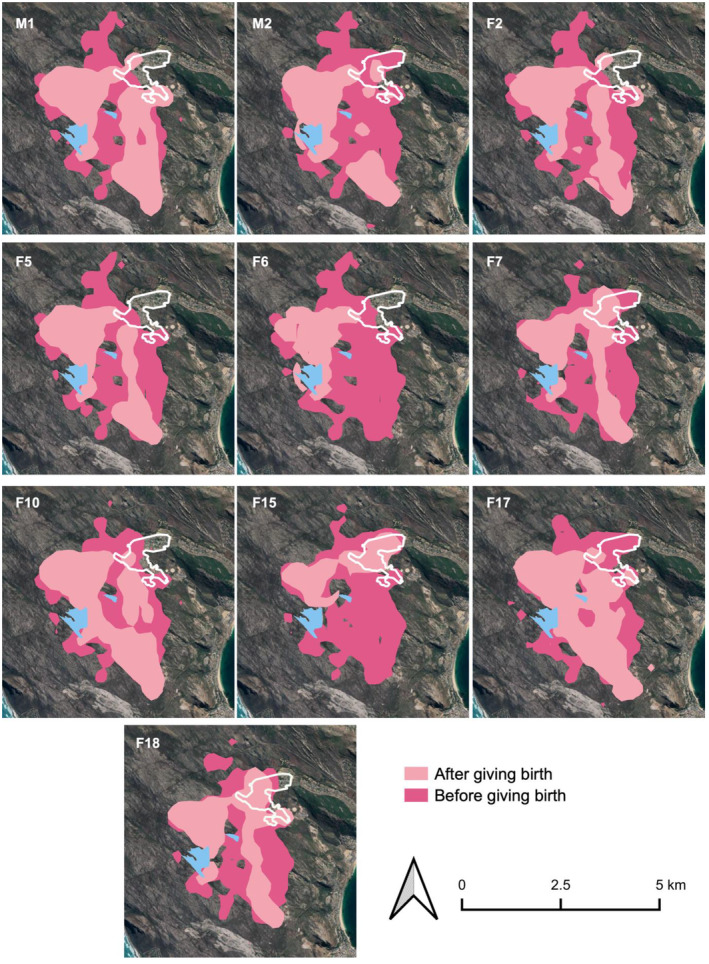
95% home range of each individual baboon before (dark pink) and after (light pink) the focal female (F13) gave birth (estimated using fixed kernel densities: Calenge, 2006). The urban polygon is indicated by a solid white line and dams are represented by light blue areas.

**FIGURE A3:**
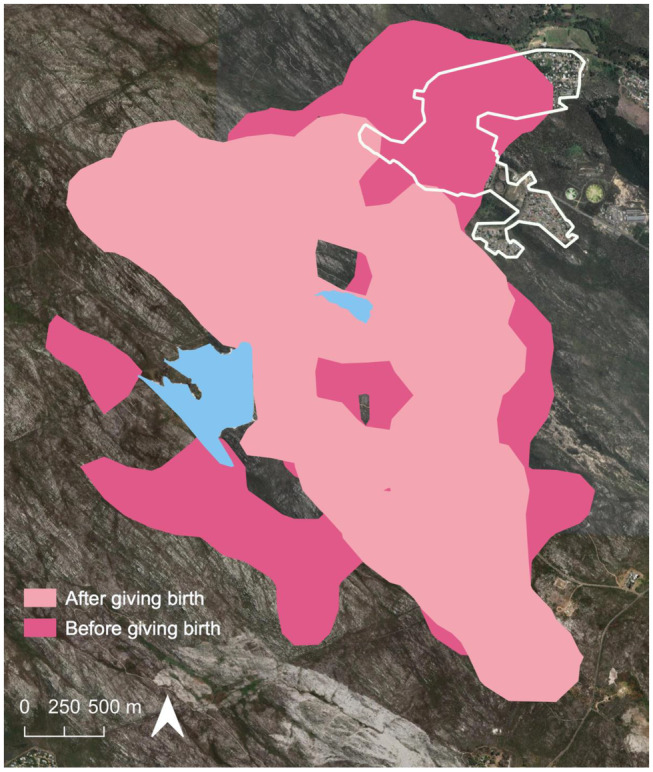
95% home range of the focal female before (dark pink) and after (light pink) giving birth (estimated using fixed kernel densities: Calenge, 2006), using a random subsample of 16 days of data before birth (to match sample size of days after birth). The urban polygon is indicated by a solid white line and dams are represented by light blue areas.

## Data Availability

All data to reproduce results are available in Dryad: https://doi.org/10.5061/dryad.z34tmpgk1.

## Methods

### GPS data

*N* = 16 adult baboons were fitted with SHOAL group in‐house constructed collars (F2HKv3), which recorded GPS positional data at 1 fix/second between 08:00 and 20:00 local time (GiPSy 5 tags; TechnoSmArt). We use “daytime” hours for the focal female and troop analyses (08:00 – 18:00 local time) as baboons were often at their urban sleepsite after 18:00. Data from 15 collars were retrieved (one collar was not found after release) and two collars failed to record GPS, resulting in individual GPS data for *n* = 13 baboons (which represented 61% of all adults in the troop), over mean ± SD of 42.77 ± 9.92 days, range = 21–54 days (Table A1). For the adult females in the troop that had GPS‐recording collars during our study, five had offspring and were lactating (ages unknown, but at least 7 months old, as all had brown adult coloration: Dezeure et al. (2021) and were present at the start of the field season), and five did not have dependent offspring. Only one female (the focal female in this study) gave birth when the GPS was recording (going from “without infant” to “with infant” on 03/09/2018).

### Urban space and home range

We defined the focal females 95% home ranges before (32 days) and after (16 days) birth, using fixed kernel densities and an *ad hoc* method for selecting the smoothing parameter, using the function “getvolumeUD” in the “adehabitat” package, R (Calenge, 2006). Within the home range, we designated urban space as the area dominated by residential buildings and surfaced roads, and drew a polygon around this using QGIS (QGIS.org, 2020). All areas outside this urban space were defined as natural space (Figure 2c). To estimate the females’ proportion of the day spent in urban space, we used the function “getRecursionsInPolygon” from the package “Recurse” in R (Bracis et al., 2018). To estimate the females’ daily distances travelled we used the function “as.ltraj” in the “adehabitat” package in R (Calenge, 2006).

### Spatial associations

We extracted individual strength and eigenvector centrality from proximity‐based social networks using the package “Spatsoc” in R (Robitaille et al., 2019) for *n* = 11 individuals, constructed before and after the focal female gave birth (mean ± SE minutes before birth: 12220 ± 76; mean ± SE minutes after birth: 1527 ± 34). Two females were removed from analyses as they did not have data after birth (F9 & F14; see Figure A1). Networks were based on times all baboons were outside the urban polygon (as proximity to humans is known to affect social networks: Bracken et al., 2022; Morrow et al., 2019) and for times when 10+ collars were active and recording synchronous data (see: Bracken et al., 2021). GPS locations were grouped temporally (1‐min intervals) and spatially (within 5 m, using the “chain rule”: Castles et al., 2014). Network edges were weighted using the simple ratio index.

**TABLE A1:** Details of the GPS recording period for each collared adult baboon (F = female, M = male in column “Individual”).

Collar	Individual	GPS start	GPS end	Total duration (days)
1	M1	30‐07‐2018	11‐09‐2018	44
2	M2	30‐07‐2018	10‐09‐2018	43
3	F1	no data		
4	F2	25‐07‐2018	13‐09‐2018	50
5	F4	no data		
6	F5	26‐07‐2018	09‐09‐2018	46
7	F6	26‐07‐2018	07‐09‐2018	44
8	F7	26‐07‐2018	09‐09‐2018	46
9	F9	26‐07‐2018	15‐08‐2018	21
10	F10	30‐07‐2018	12‐09‐2018	45
11	F13	02‐08‐2018	24‐09‐2018	54
12	F14	02‐08‐2018	25‐08‐2018	24
13	F15	26‐07‐2018	07‐09‐2018	44
14	F17	02‐08‐2018	19‐09‐2018	49
15	F18	26‐07‐2018	09‐09‐2018	46
16	F19	no data		

*Note*: One collar (F1) collar was not retrieved after drop‐off. Two GPS (F4 and F19) failed to record GPS data. Focal female (F13) indicated in red.
